# Self‐Reported Perceptions of Patients and Staff on Participation and Verbal and Social Interactions in High‐Security Forensic Psychiatric Care in Sweden

**DOI:** 10.1111/jpm.13105

**Published:** 2024-09-15

**Authors:** Andreas Söderberg, Märta Wallinius, Christian Munthe, Ulrica Hörberg, Mikael Rask

**Affiliations:** ^1^ Department of Health and Caring Sciences Linnaeus University Växjö Sweden; ^2^ Department of Clinical Sciences Lund Lund University Lund Sweden; ^3^ Department of Philosophy, Linguistics and Theory of Science University of Gothenburg Gothenburg Sweden

**Keywords:** forensic psychiatry, mental health settings, quantitative methodology

## Abstract

**Introduction:**

Studies suggest that experiences of patient participation, as described by both patients and staff, are associated with a significant caring relationship of high quality.

**Aim:**

This study aimed to investigate staffs' and patients' self‐reported perceptions on participation and the frequency and importance of verbal and social interactions in high‐security forensic psychiatry.

**Method:**

The questionnaire Verbal and Social Interactions (VSI) was used together with the subscale *Participation* from Quality in Psychiatric Care (QPC). The study was conducted at a large forensic psychiatric clinic in Sweden.

**Results:**

Staff and patients rated the frequency of VSI differently while reporting the same perceived degree of patient participation. All categories of VSI were significantly correlated with perceived level of participation with medium or small effect size for the patients.

**Discussion:**

Patients' perceived participation seems to depend on verbal and social interaction within the specific categories ‘*Showing interest in the patients’ feelings*, *experiences*, *and behavior’* and *‘Helping the patients establish structure and routines in their everyday life’*. There was, however, a *negative* association for the latter.

**Implications for Practice:**

The results give a better understanding of what kind of interactions that affect patients' perceived level of participation.

**Relevance Statement:**

Patient participant is a prioritised area for development in forensic psychiatry. The study contributes to a better understanding of what types of interactions that affect the perceived level of participation, while at the same time, it shows important similarities and differences between patient and staff perspectives.

**Summary**

**What Is Known on the Subject?**

- Previous studies carried out in forensic psychiatric care indicate that participation is a complex concept in institutions where compulsory treatment and deprivation of liberty are core elements.
- Studies have shown that experiences of patient participation, as described by both patients and staff, are associated with a caring relationship of a high quality. Such a caring relationship is defined by, for example, good communication, development of trust and staff having an encouraging attitude towards the patients.

**What the Paper Adds to Existing Knowledge?**

- The question of how, and in which way, different aspects of caring relationships relate to the experience of patient participation remains unclear, especially in a forensic psychiatric care context.
- Research in forensic psychiatric care tends to either focus on the care provided in wards with lower levels of security or includes all security levels. Studies highlighting caring relationships and patient participation in the context of high‐security units have not been undertaken previously and tend to target only staff or patients, not both.

**What Are the Implications for Practice?**

- The most important result in this study was the association between the frequency of interactions in the category ‘*Showing interest in the patients’ feelings*, *experiences and behavior’* and patient participation.
- It is also noted that that staff and patients rated the frequency of verbal and social interactions differently, while reporting the same perceived degree of participation.

## 1 Introduction

Previous studies carried out in forensic psychiatric care indicate that participation is a complex concept in an institution where compulsory treatment and deprivation of liberty are core elements (Söderberg, Wallinius, and Hörberg 2020; Söderberg et al. 2022; El‐Alti, Sandman, and Munthe 2022).

Selvin et al. (2019) performed a repeated cross‐sectional survey, measuring dimensions of quality of care (Encounter, Participation, Discharge, Support, Secluded Environment, Secure Environment, Forensic Specific), with a data collection at two forensic psychiatric clinics each year from 2011 to 2014. They found that the dimension of patient participation was rated lowest or second lowest of all dimensions across all the years, by both patients and staff. Similar results (lowest score for participation, rated by patients) was found in studies with the same instrument in 2009 in Sweden (Schröder and Lundqvist 2013; Schröder, Ågrim, and Lundqvist 2013; Lundqvist and Schröder 2015) and in Denmark (Schröder et al. 2016).

Studies have also shown that the experience of patient participation, as described by both patients and staff, is associated with a caring relationship that has high quality. Such a caring relationship is defined by, for example, good communication, development of trust and the staff having an encouraging attitude towards the patients (Söderberg, Wallinius, and Hörberg 2020; Söderberg et al. 2022; Selvin et al. 2016; Sahlsten et al. 2008). Further knowledge is, however, needed to clarify the existence and more precise nature of this caring relationship and how it may influence the experience of patient participation in forensic psychiatry.

A well‐cited, umbrella review article on factors influencing the caring relationship between nurses and patients indicated that both nurses and patients had expectations regarding the caring relationship and that these expectations need to be met if the caring relationship is to be viewed as positive. Patients assessed the quality of the relationship through the alignment of values displayed around caring behaviours and attitudes demonstrated by the nurse (Wiechula et al. 2016). Previous research shows that staff and patients rate perceptions of the frequency and importance of nursing care interactions differently in forensic psychiatry (Rask and Brunt 2006) and for psychiatric outpatients (Rask et al. 2022). However, the question of how, and in which way, different aspects of caring relationships relate to the experience of patient participation remains unclear, especially in a forensic psychiatric care context. Research in forensic psychiatric care tends to either focus on the care provided in wards with lower levels of security or includes all security levels. Studies highlighting caring relationships and patient participation in the context of high‐security units have not been undertaken previously. Moreover, many studies target only staff or patients.

## 2 Aim

The aim of this study was thus to investigate the staffs' and the patients' self‐reported perceptions of patient participation and the frequency and importance of verbal and social interactions in high‐security forensic psychiatric care, with the following, specific questions:

- Do patients and staff rate patients' participation in care and frequency and importance of the staffs' verbal and social interactions in high‐security forensic psychiatric care differently? If so, in which ways do they differ?
- Do perceptions of patient participation and the frequency and importance of verbal and social interactions correlate in high‐security forensic psychiatric care? If so, in which aspects?
- Is the patients' perceived level of participation dependent on the staffs' verbal and social interactions? If so, in which way?

## 3 Materials and Methods

### 3.1 Preregistration

This study, with planned sample size, included variables, hypotheses and planned analyses, was preregistered on Aspredicted (https://aspredicted.org/c262k.pdf) prior to data collection and analysis. The study was approved by the Swedish Ethical Review Authority.

### 3.2 Settings

There are 26 forensic psychiatric settings with a total of ~1270 inpatient beds in Sweden (Swedish National Forensic Psychiatric Register 2022), with 30% of the inpatient beds being classified at security level 3, 7%–8% at Level 1, and the rest at Level 2 (Swedish Association of Local Authorities and Regions 2022). The study sample thus constituted ~10% of the total population of forensic inpatients at security levels 1 and 2 in Sweden.

The study was conducted at a large forensic psychiatric clinic in Sweden, with ~120 inpatient beds distributed over three security levels; security levels: 1 (very high), 2 (high) and 3 (satisfactory) (The National Board of Health and Welfare's regulations Concerning Security in Health Care Facilities 2017). This study focussed on high security, forensic psychiatric care, including only security levels 1 and 2, providing a total of 87 eligible inpatients.

### 3.3 Participants

Patient participants were admitted to forensic psychiatric care in accordance with the Forensic Mental Care Act (1991, 1129) or treated within forensic psychiatry care while serving a prison sentence (due to poor mental health). The inclusion criterion for the patient participation was inpatient care at one of the clinic's seven wards at security levels 1 or 2. Patients with insufficient Swedish‐language skills to complete the self‐report questionnaires were excluded from the study, as well as patients who were assessed as ineligible due to their psychiatric status (e.g., acute psychosis and severe intellectual disorder) by the ward's psychiatrist. Background variables collected for patients were as follows: sex, age, previous contacts with psychiatry, time spent in forensic psychiatric care and current security level.

The inclusion criteria for staff participants were as follows: at least 3 months of work experience at a ward on the clinic (i.e., registered nurses, nurse assistants with a mental health education, social pedagogues and behavioural scientists, both the latter groups have a higher education and work at the ward with a with an extended responsibility of social activities). Professionals affiliated to the wards such as psychiatrists, social workers and psychologists were not included. Background variables collected for staff were: sex, age, profession and length of employment at the clinic.

### 3.4 Data Collection

All patients fulfilling the inclusion criteria were invited to participate. A total of eight patients were excluded due to psychiatric status, and five were excluded due to insufficient language skills, thus generating an eligible sample of 74 patients. Seven ward liaisons, one at each ward, who were specifically trained by the first author for the task, informed eligible patients of the study and collected written consent and the completed questionnaires. A total of 42 patients participated (57% participate rate).

To match the patient group to a staff group, the first author distributed information and questionnaires to 100 ward staff at the beginning of their shift during a 24‐h day (dayshift, evening shift and nightshift). A total of 66 staff participated with 66% participation rate (Tables 1 and 2).

**TABLE 1:** Characteristics of the study group—Patients.

	*N*	%
Sex		
Female	3	7.1
Male	39	92.9

	Mean	Range
Age	34.3	21–59

	*N*	%
Previous contact with psychiatry		
No	10	23.8
Yes, inpatient	22	52.4
Yes, only outpatient	10	23.8
Time spent in FPC		
0–6 month	2	4.8
6 month–2 year	9	21.4
2–5 year	11	26.2
5–10 year	9	21.4
More than 10 year	11	26.2
Currently security level		
Security level 1	14	33.3
Security level 2	28	66.7

**TABLE 2:** Characteristics of the study group—Staff.

	*N*	%
Sex		
Female	33	50
Male	33	50

	Mean	Range
Age	39	19–63

	*N*	%
Education		
Registered Licensed Nurse	17	25.8
Nurse assistant	42	63.6
Other	7	10.6
Length of employment		
0–6 month	2	3.0
6 month–2 year	14	21.2
2–5 year	6	9.1
5–10 year	23	34.8
More than 10 year	21	31.8

### 3.5 Measurements

The instruments used were the subscale *Participation* from the questionnaires Quality in Psychiatric Care‐Forensic In‐Patient (QPC‐FIP) and Quality in Psychiatric Care‐Forensic In‐Patient Staff (QPC‐FIPS) (Schröder, Ågrim, and Lundqvist 2013; Schröder and Lundqvist 2013) and the questionnaire Verbal and Social Interactions (VSI; Rask, Brunt, and Fridlund 2008).

#### 3.5.1 QPC

The first version of the QPC questionnaire (Schröder, Wilde Larsson, and Ahlström 2007; Schröder et al. 2010) was derived from the results of an interview study with a phenomenographic approach, with in‐ and outpatients from psychiatric care (Schröder, Ahlström, and Larsson 2006). The QPC‐FIP and QPC‐FIPS were then developed from the original QPC and adjusted to the forensic psychiatric context. QPC measures several dimensions associated with quality of care, one of which is participation (Schröder, Ågrim, and Lundqvist 2013; Schröder and Lundqvist 2013). All the items are related to questions that are formulated in terms of ‘I feel like’ with a response on a 4‐point Likert scale (1—totally disagree; 4—totally agree). It was also possible to answer ‘not applicable’. QPC‐FIP and QPC‐FIPS have previously demonstrated good psychometric properties (Schröder, Ågrim, and Lundqvist 2013; Schröder and Lundqvist 2013), with internal consistency for the whole QPC‐FIP at *α* = 0.98, and for the subscale *Participation* at *α* = 0.93. In this study, one item (recognise signs of deterioration) was removed due to the low connection to the concept of participation and its focus being on a medical aspect of the care. Seven items from the subscale *Participation* were thus used in this study.

#### 3.5.2 VSI

The VSI instrument is based on a philosophical and theoretical foundation (Rask and Brunt 2007). The authors (2007) created a model of how six categories of nursing interactions interlink. Inspired by philosophers such as Merleau‐Ponty (1908–1961) and his description of ‘the between’ in interpersonal relationships (1995), Rask and Brunt (2007) developed a model of VSI in the nurse–patient relationship in forensic psychiatric nursing care. The model was also inspired by the literature and nursing theories of Peplau (1988), as well as their own research in the field (Rask and Levander 2001). The questions in VSI for the patients are formulated as, for example: *The staff show you that they are honest*. The same question for the staff is formulated as: *I show the patient that I am honest*. VSI also measures the same question but from the perspective of how important it is, for example; *The staff show you that they are honest*—*How important is this?* The responses are on a 4‐point Likert scale (1—not at all; 4—to a very high degree). There is no response alternative ‘not applicable’ as in the subscale from QPC‐FIP.

VSI consists of 21 items, divided into three subscales (categories); *Inviting the patient to establish a relationship* (8 items); *Showing interest in the patients' feelings*, *experiences and behavior* (7 items) and *Helping the patients to establish structure and routines in their everyday life* (6 items). The internal consistency of the questionnaire has previously been reported at *α* = 0.91 (Rask, Brunt, and Fridlund 2008).

### 3.6 Data Analysis

All analyses were performed in IBM SPSS Statistic version 28.0.1.1(14). Visual inspection of the data using histograms demonstrated non‐normal distribution of data. Missing data were replaced with the mean for the corresponding item. The comparison of the means of each subscale/category (*showing interest in the patients' feelings*, *experiences*, *and behavior*; *helping the patients to establish structure and routines in their everyday life*; *participation*) is presented in Table 3. An independent sample Mann–Whitney *U*‐Test was used to compare mean ranks between patients and staff. Percentages were calculated for the scores 3 and 4 (high and very high) for both the frequency and importance of VSI as in previous publications on the instrument (Brunt and Rask 2018). Percentages from the QPC‐FIP degree of agreement (agree for the most part, 3; totally agree, 4) were calculated for the subscale *Participation* in the same way as for VSI (Table 4). Correlations between participation and the frequency and importance of VSI were investigated using Spearman's rho (Tables 5 and 6). A multiple stepwise linear regression was performed to test whether the patients' perceived level of participation was dependent on the perceived level of frequency of any of the categories in VSI (Table 7). In a first step, the model controlled for background variables and in a second for VSI categories. Multicollinearity was not an issue in the models considering the variance inflation factor and tolerance values presented.

**TABLE 3:** Comparison of the perceptions of patients and staff of the frequency and importance of verbal and social interactions and of patient participation.

	Patient frequency	Staff frequency	*p*	Patient importance	Staff importance	*p*
	Mean (SD)	Mean (SD)		Mean (SD)	Mean (SD)	
VSI—total score	2.54 (0.8)	3.10 (0.4)	< 0.001***	3.08 (0.8)	3.34 (0.5)	0.339
Inviting the patient to establish a relationship	2.69 (0.8)	3.42 (0.6)	< 0.001***	3.42 (0.8)	3.59 (0.5)	0.491
Showing interest in the patients' feelings, experiences and behavior	2.47 (0.9)	2.75 (0.6)	0.077	3.00 (0.9)	3.02 (0.6)	0.769
Helping the patients to establish structure and routines in their everyday life	2.42 (1.1)	3.09 (0.7)	0.001**	2.72 (1.1)	3.38 (0.6)	0.006**

	Patient	Staff				
	Degree of agreement	Degree of agreement				
	Mean (SD)	Mean (SD)				
Participation (QPC‐FIP)	2.61 (0.9)	2.81 (0.7)	0.540			

*p < 0.05, **p < 0.01, and ***p < 0.001.

**TABLE 4:** Valid percentage (scores 3 and 4) of perceptions of patients and staff of the frequency and importance of verbal and social interactions and of patient participation.

	Patient frequency	Staff frequency	Patient importance	Staff importance
Inviting the patient to establish a relationship				
The staff show you that they are honest	54.8	93.9	83.3	93.8
The staff show the patients that you can believe in them	61.9	93.9	81	93.8
The staff show you that you can trust them	61.9	97	85.7	96.9
The staff show you that they care about you	48.8	87.2	76.6	96.9
The staff show you that you can feel safe with them	61.9	97	81	95.4
The staff show you that they are true to their word	57.1	97	85.7	96.9
The staff show you that they want to get to know you	59.5	81.8	81	86.2
The staff show you that they want to make contact with you.	50	72.7	81	81.5
Showing interest in the patients' feelings, experiences and behaviour				
The staff talk to you about your feelings about other people	45.2	63.6	64.3	78.5
The staff describe to you how they perceive your behaviour	64.3	69.2	76.2	73.8
The staff talk to you about your feelings	45.2	78.5	61.9	82.8
The staff talk to you about how you think about other people	41,5	66.7	68.3	78.5
The staff encourage you to talk about why you are on the ward	40.5	53	57.1	66.2
The staff encourage you to talk about negative experiences prior to and while on the ward	38.1	43.9	57.1	61.5
The staff talk to you about your behaviour when you are with other people	47.6	69.2	71.4	72.1
Helping the patients to establish structure and routines in their everyday life				
The staff describe to you why it is important for you to keep your things in good order	57.1	69.7	59.5	83.1
The staff encourage you to maintain your personal hygiene	45.2	80.3	66.7	95.4
The staff encourage you to keep your belongings in good order	52.4	77.3	57.1	89.2
The staff describe to you why it is important for you to keep regular sleeping hours	45.2	78.8	47.6	90.8
The staff encourage you to take care of your finances	45.2	59.1	52.4	76.9
The staff describe to you why it is important to eat regular meals	45.2	72.7	50	90.8

Participation (QPC‐FIP)	Patients degree of agreement	Staff degree of agreement		
Patients have influence over their care	50	53		
Patients' view of the right care is respected	47.6	51.5		
Patients take part in decision‐making about their care	48.8	54.5		
Benefit drawn from the patient's earlier experience of treatment	61.0	60.9		
Patients informed in a way that they understand	64.3	84.6		
Patients have knowledge about their mental troubles	61.0	72.3		
Patients receive information about treatment alternatives	52.5	50.8		

**TABLE 5:** Spearman's rho for patients. Correlations of the perceptions of the frequency and importance of verbal and social interactions and of patient participation.

		Inviting the patient to establish a relationship		Showing interest in the patients' feelings, experiences and behaviour		Helping the patients to establish structure and routines in their everyday life		VSI – total score		Participation (QPC‐FIP)
		Frequency	Importance	Frequency	importance	Frequency	importance	Frequency	importance	
Inviting the patient to establish a relationship	Frequency	1.000								
	Importance	0.126	1.000							
Showing interest in the patients' feelings, experiences and behaviour	Frequency	0.783**	0.248	1.000						
	Importance	0.223	0.698**	0.478**	1.000					
Helping the patients to establish structure and routines in their everyday life	Frequency	0.549**	0.122	0.603**	0.387*	1.000				
	Importance	0.224	0.402**	0.480**	0.734**	0.660**	1.000			
VSI – total score	Frequency	0.894**	0.174	0.913**	0.410**	0.799**	0.505**	1.000		
	Importance	0.247	0.718**	0.507**	0.933**	0.503**	0.844**	0.468**	1.000	
Participation (QSP‐FIP)		0.681**	0.204	0.731**	0.201	0.368*	0.134	0.677**	0.221	1.000

**Correlation is significant at the 0.01 level (two‐tailed).

*Correlation is significant at the 0.05 level (two‐tailied).

**TABLE 6:** Spearman's rho for staff. Correlations of the perceptions of the frequency and importance of verbal and social interactions and of patient participation.

		Inviting the patient to establish a relationship		Showing interest in the patients' feelings, experiences and behaviour		Helping the patients to establish structure and routines in their everyday life		VSI – Total score		Participation (QPC‐FIP)
		Frequency	Importance	Frequency	importance	Frequency	importance	Frequency	importance	
Inviting the patient to establish a relationship	Frequency	1.000								
	Importance	0.667**	1.000							
Showing interest in the patients' feelings, experiences and behaviour	Frequency	0.349**	0.271*	1.000						
	Importance	0.284*	0.487**	0.706**	1.000					
Helping the patients to establish structure and routines in their everyday life	Frequency	0.562**	0.234	0.251*	0.127	1.000				
	Importance	0.491**	0.420**	0.309*	0.401**	0.761**	1.000			
VSI—total score	Frequency	0.781**	0.449**	0.710**	0.498**	0.787**	0.687**	1.000		
	Importance	0.550**	0.731**	0.614**	0.861**	0.409**	0.709**	0.680**	1.000	
Participation (QSP‐FIP)		0.236	0.122	0.205	0.070	0.394**	0.359**	0.342**	0.187	1.000

**Correlation is significant at the 0.01 level (two‐tailed).

*Correlation is significant at the 0.05 level (two‐tailed).

**TABLE 7:** Multiple linear regression analysis of associations between VSI scales, background variables (independents) and QPC subscale participation (dependent).

	Participation (dependent) Model 1		*p*
	Standardised coefficients beta	*t*	
Age	−0.243	−1.340	0.188
Previous contact in psychiatry	−0.185	−1.176	0.247
Time spent in FPC	−0.150	−0.829	0.413
Currently security level	0.012	−0.075	0.941
*R* ^2^	0.138		
Adjusted *R* ^2^	0.045		

	Participation (dependent) Model 2		*p*
	Standardised coefficients beta	*t*	
Age	−0.124	−1.031	0.310
Previous contact in psychiatry	−0.175	−1.700	0.098
Time spent in FPC	−0.198	−1.398	0.171
Currently security level	0.026	0.251	0.804
VSI dimensions			
Inviting the patient to establish a relationship	0.302	1.688	0.100
Showing interest in the patients' feelings, experiences and behaviour	0.626	3.149	0.003**
Helping the patients to establish structure and routines in their everyday life	−0.309	−2.049	0.048*
*R* ^2^	0.67		
Adjusted *R* ^2^	0.60		

*p < 0.05, **p < 0.01, and ***p < 0.001.

## 4 Results

Most of the patient participants were men, and a majority of these reported that they had been cared for in the forensic psychiatric unit for more than 2 years. One‐third of the patient participants were currently placed in security class 1, and the rest in security class 2 (Table 1). There were an equal number of men and women among the staff participants, and most of them had worked for more than 2 years. A majority of the staff participants were licensed nurse assistants, a quarter were registered nurses, and the rest had another professional title, that is, social pedagogues and behavioural scientists (Table 2).

### 4.1 Patient Participation and the Staff's Verbal and Social Interactions

#### 4.1.1 Participation

There was no statistically significant difference between how patients and staff rated patient participation in high‐security, forensic psychiatric care (*z* = −0.612, *p* = 0.540) (Table 3). The percentages were very similar in the comparison of the patient and staff groups who had rated a 3 or a 4 (agree for the most part and totally agree). For example, 50% in both groups rated a 3 or a 4 for the question *‘Patients have influence over their care’*. An exception to the pattern presented above was for the item: *‘Patients are informed in a way that they understand’*, where 64.3% of the patients rated a 3 or a 4 compared with 84.6% of staff (Table 4). Cronbach's *α* for Participation was *α* = 0.87 (staff) and *α* = 0.90 (patient).

#### 4.1.2 Staff's Verbal and Social Interactions

Patients rated the frequency of the staff's VSI at a significantly lower level than the staff did (*z* = −3.758, *p* =< 0.001). Both staff and patients rated ‘Inviting *the patient to establish a relationship’* as the most commonly occurring category of interactions. The staff rated *‘Showing interest in the patients' feelings*, *experiences and behavior’ as* the least commonly occurring category of interactions, while patients rated the category *‘Helping the patients to establish structure and routines in their everyday life’* as the least commonly occurring. There was no significant difference between how patients and staff rated interactions in the category *‘Showing interest in the patients' feelings*, *experiences and behavior’* in the comparison of mean ranks (*z* = −1.771, *p* = 0.077). However, the staff rated their interactions for the categories *‘Inviting the patient to establish a relationship’* (*z* = −4.700, *p* =< 0.001) and *‘Helping the patients to establish structure and routines in their everyday life’* (*z* = −3.237, *p* = 0.001) as occurring more often than the patients did at a significant level.

There was no significant difference between how patients and staff rated the importance of verbal and social interactions for the total VSI (*z* = −0.956, *p* = 0.339). Both patients and staff reported *‘Inviting the patient to establish a relationship’* as the most important category of interactions. The only significant difference in terms of the importance of the interactions was reported for *‘Helping the patients to establish structure and routines in their everyday life’*, where staff reported these as more important than the patients did (*z* = −2.769, *p* = 0.006).

The internal consistency for the whole VSI questionnaire was *α* = 0.89 (staff) and *α* = 0.96 (patients). ‘*Inviting the patient to establish a relationship’* was *α* = 0.88 (staff) and *α* = 0.94 (patients), ‘Showing interest in the patients' feelings, experiences and behavior’ was *α* = 0.80 (staff) and *α* = 0.91 (patients), and *‘Helping the patients to establish structure and routines in their everyday life’* was *α* = 0.88 (staff) and *α* = 0.93 (patients).

##### 4.1.2.1 Inviting the Patient to Establish a Relationship

A majority of both staff and patients rated the importance of this category at a high or very high level. However, there was a large difference between the perceptions of the patients and staff in terms of the frequency with the largest disparities for three questions; *‘The staff show you that they are honest*’ where 54.8% of patients rated this at a high or very high level, in comparison to the staff's 93.9%. The corresponding figures for *‘The staff show you that they care about you*’ were 48.8% for the patients and 87.2% for the staff and for *‘The staff show you that they are true to their word’* were 57.1% for the patients and 97% for the staffs (Table 4).

##### 4.1.2.2 Showing Interest in the Patients' Feelings, Experiences, and Behaviour

A smaller percentage of both patients and staff rated this category at a high or very high level (a 3 or a 4) for both the frequency and the importance. The same pattern of differences between the two groups as described above emerged also for this category, where the percentage of staff who rated a high or very high level was greater than for the patient group, even if the difference for most items was smaller than in the comparison above (Table 4).

##### 4.1.2.3 Helping the Patients to Establish Structure and Routines in Their Everyday Life

The groups differed in terms of both the frequency and the importance of the interactions in this category. For example, 45.2% of the patients rated a 3 or a 4 for the frequency on the question concerning personal hygiene in comparison with 80.3% by the staff. The difference in the rated importance for the same question was 66.7% for the patients (high or very high degree of importance) in comparison with 95.4% for the staff. A similar result was found for the question concerning keeping regular sleeping hours where only 47.6% of the patients rated it to be important to a high or very high degree compared with 90.8% of the staff (Table 4).

### 4.2 Correlations Between Patient Participation and the Frequency and Importance of Verbal and Social Interactions

#### 4.2.1 Patients

Spearman's rank correlation was calculated to assess the relationship between the perceived patient participation and the staff's verbal and social interactions. Participation was significantly correlated with the VSI total at *p* < 0.001 with a medium effect size (*r* = 0.68). The category of actions with the highest correlation to participation was *‘Showing interest in the patients' feelings*, *experiences and behavior’* (*r* = 0.731; *p* < 0.001), the second highest was *‘Inviting the patient to establish a relationship’* (*r* = 0.681; *p* < 0.001) and the lowest correlation was for *‘Helping the patients to establish structure and routines in their everyday life’* (*r* = 0.368; *p* = 0.017). All three categories of interactions were correlated with patient participation at a significant level. However, there was no significant relationship between the level of importance of the verbal and social interactions and the perceived level of participation in their care as rated by the patients (*r* = 0.221; *p* = 0.159) (Table 5).

#### 4.2.2 Staff

Patient participation was, according to the staff, significantly correlated with VSI total at *p* = 0.005 with a medium effect size (*r* = 0.34). However, this association was solely maintained by a significant correlation (*r* = 0.39; *p* = 0.001) between patient participation and the category *‘Helping the patients to establish structure and routines in their everyday life’*. There was no significant relationship between the level of importance of the verbal and social interactions and patient participation as rated by the staff, except for the category *‘Helping the patients to establish structure and routines in their everyday life’* (*r* = 0.359; *p* = 0.003) (Table 6).

### 4.3 Relationship Between Patients' Perceived Level of Participation and Staff's Verbal and Social Interaction

Associations between the VSI categories and background variables and *Participation* were tested in the multiple linear regression analysis (Table 7).

Only the associations between background variables (age, previous contact in psychiatry, time spent in forensic psychiatric care, currently security level) and patient participation were tested in Model 1. None of the background variables showed a significant association to patient participation and the variance explained by the model was only ~5%. The VSI categories were added to the analysis in Model 2, resulting in an explained variance of 60%. *‘Showing interest in the patients’ feelings*, *experiences and behavior’* (*β* = [0.626], *p* = [0.003]) predicted patient participation at a higher level than *‘Helping the patients to establish structure and routines in their everyday life’ did* (*β* = [−0.309], *p* = [0.048]). The category *‘Inviting the patient to establish a relationship’* did not predict patient participation (*β* = [0.302], *p* = [0.100]) at a significant level. None of the background variables predicted patient participation, as seen in Model 1.

## 5 Discussion

The aim of this study was to investigate the staff's and patients' self‐reported perception of participation and the frequency and importance of verbal and social interactions in high‐security forensic psychiatry. The main findings of this study were that staff and patients rated the frequency of verbal and social interactions differently, while reporting the same perceived degree of participation. All the categories of the verbal and social interactions were, in the patients' responses, correlated with the perceived level of participation at a significant level. Finally, the patients' perceived participation appears to depend on the verbal and social interactions found in the categories *‘Showing interest in the patients’ feelings*, *experiences and behavior’* and *‘Helping the patients to establish structure and routines in their everyday life’*.

The most important result in this study was the association between the frequency of interactions in the category ‘*Showing interest in the patients’ feelings*, *experiences and behavior’* and patient participation, the strength of association was medium. An example of how the items in this category are formulated is as follows: ‘The staff talk to you about your behavior when you are with other people’. There was no significant association between the frequency of interactions in the category *‘Inviting the patient to establish a relationship’* and patient participation. This suggests that the presence of *some* form of caring relationship is not what affects patient participation that the patients themselves are aware of and view as important, but instead what the staff actually do and communicate in the course of a caring relationship. Staff who display an interest in patients' feelings and thoughts are more likely to positively affect the patients' perception of patient participation. Furthermore, the results suggest the importance of not only talking about feelings and thoughts but also responding to patients' behaviours. Statements such as ‘the staff can tell me both when I am right and when I am wrong’ are common in qualitive, interview‐based research when patients explain participation. Such statements aim to describe the importance of feeling humanised in various ways, a feeling of being treated as an equal (Söderberg et al. 2022). The result in this study is in line with those types of statements and their association with patient participation.

Nyman et al. (2022) emphasise the benefits of involving forensic psychiatric patients in their risk assessments, both in terms of providing patients with an opportunity to better understand their own care process and that patients probably are more capable of participating in the assessments than expected. This is in line with the results in a qualitative study made by El‐Alti, Sandman, and Munthe (2022), where the staff's perception of the patients' agential capacity is often high, even though staff also talk of a small number of patients in forensic psychiatric settings as having a low capacity level and poor insight into their own conditions. The comparison of the perceptions of patients and staff of the frequency and importance of verbal and social interactions in this study suggests that the two groups do not differ significantly in the category where this kind of questions is included. They rated the importance of ‘*Showing interest in the patients' feelings*, *experiences and behavior’* at a similar level. It is notable that this was the only category in VSI where the patients and staff rated the frequency of the interactions at a similar level.

It is also noteworthy that the staff rate a higher level of importance than the patients do in the performance of the interactions in the VSI category ‘*Helping the patients to establish structure and routines in their everyday life’*, while the multiple regression analysis showed a significant, *negative* association between patients' perceived degree of participation and the frequency of interventions for this VSI category. From a clinical perspective, this could mean that patients perceive a lower degree of participation when being reminded to clean their room, keep their things in order or when staff ask them to maintain a good personal hygiene, while staff maintain the notion that these actions are important factors for enhanced patient participation. Helping patients to maintain a good structure in life is important from many perspectives, for example, we know that the circadian rhythm affects mental health (Walker et al. 2020). The results in this study do not entail that staff should stop helping patients to establish good routines and structure in their lives. However, there are reasons for staff to critically assess any notion that they are thereby necessarily making the care more participatory. This is a good example of a goal conflict in forensic psychiatric care (Söderberg et al. 2022) and of the observation that forensic psychiatric staff have a tendency to view any adherence to care routines as patient participation (El‐Alti, Sandman, and Munthe 2022). The question can thus be raised about *how* staff interact and if the conflicting goals could be better aligned by staff acting and communicating differently with patients. For example, a comparison can be made of the patients' experience when staff command them to clean their room with their experience when staff remind them to maintain their own well‐being by cleaning their room. Examples, such as those presented above, about *how* staff communicate are associated with the meaning of good caring relationships between staff and patients (Magnusson, Axelsson, and Lindroth 2020).

The mean score for the subscale participation was 2.61 for patients and 2.81 for staff. These results are very similar to those in a study sample from 2009 and 2011 when the QPC questionnaire was completed by 66 patients and 202 staff members from 12 forensic units in Sweden (Lundqvist and Schröder 2015). This indicates that despite 10 years of work to increase patient participation in Sweden, with projects such as ‘Better care less Coercion’ (National Board of Health and Welfare 2023), the rating of the perceived degree of participation in forensic psychiatric care, based on the QPC subscale *Participation*, has not been impacted, at least not in a significant way. However, it is difficult to draw a conclusion from this, as the units are different and there are different security levels in both studies. Nevertheless, this could indicate that patient participation in this type of context is at a lower level either due to the inherent nature of a forensic psychiatric unit, where the patients are placed there against their will thus making it less likely that they rate participation at a higher level, or it is because the efforts to increase patient participation have been ineffective.

### 5.1 Strength and Limitations

One of the strengths of this study was that both the staff and the patients were from the same clinic and the same wards and that they completed the questionnaires during the same time period. Another strength was that the patients constituted a total sample, with a participation rate similar to that in other studies in this field (Laporte et al. 2021). However, the current sample is smaller in comparison with other studies using participants from several clinics, thus constituting a limitation. On the other hand, using different units in a study can generate other limitations in the overall interpretation of results since differences have been found between units (Swedish National Forensic Psychiatric Register 2022). It should be noted that the size of the sample still means that the regression should be seen as exploratory, which is a limitation in the study.

Regarding generalisability from this study, the study clinic had an overrepresentation of patients from security level 1 compared with corresponding clinics in the rest of Sweden. However, the security level was not associated with the degree of patient participation when controlled for in the multiple regression, which is something that would need to be corroborated in larger studies over several units.

Another possible limitation concerned the psychometric properties of the used instruments. Cronbach's alpha for the total VSI in this study was > 0.90 for the patient group. According to Tavakol and Dennick (2011), such high values (> 0.90) may suggest redundancies and show that the number of items in the instrument should be reduced. However, Cronbach's alpha was only > 0.90 for the patients but not the staff. This should be investigated in further studies prior to alterations of the VSI being made.

Pedersen et al. (2021) made a brief overview of the literature in four different forensic mental health journals to investigate the transparency of reporting in terms of recruitment and representativeness and made suggestions for improving reporting practices: (1) a more explicit description on how the sample relates to the overall population, (2) nonparticipants are described as much as possible and (3) applying power analysis and preregistration. No power analysis was performed in the current study since a total sample of patients was sought. We did, however, carry out a preregistration. The preregistration has been a support during the study and hopefully facilitates for the reader to understand and evaluate the study.

According to the preregistration, if a participant has seemingly responded without assessing each item on its merit, for example, has used the same response category for all the items in questionnaire, then the participant's whole questionnaire would be excluded. This turned out to be difficult when, for example, patients used the response category with the lowest value for all the questions and were perceived to have answered truthfully. While this was not a common occurrence in this study, the reader should know that no questionnaires were excluded. Furthermore, the deviations from the preregistration is; No boxplots and no multiple linear regression analysis on the staff's questionnaires was performed. This decision was based on the conclusion that such an analysis did not correspond to the registered aim of our study. A Spearman's rank correlation was instead calculated to assess the relationship between the perceived participation and the verbal and social interactions, for both patients and staff, which was not referred to in the preregistration but was considered important for the overall study purpose.

## 6 Implications for Practice

Forensic psychiatric services can benefit from acknowledging and searching for approaches to deal with forensic psychiatry's various goal conflicts that all stem from the balance between community protection and caring. The most important cause for patients to experience a higher degree of participation seems to depend on being treated in a human way—as a person with feelings and experiences and behaviours that matter.

## Conflicts of Interest

The authors declare no conflicts of interest.

## Data Availability

Research data are not shared.
